# Photothermal Polarimetric Nanoscopy: An Emerging Technique for Fingerprinting Minerals of Extraterrestrial Origin

**DOI:** 10.1021/acsearthspacechem.5c00124

**Published:** 2025-09-11

**Authors:** T. Shay, K. Hinrichs, S.G. Pavlov, N. Stojanovic, I. Weber, A. Morlok, M. Gensch

**Affiliations:** † ASML Berlin GmbH, Waldkraiburger Str. 5, Berlin 12347, Germany; ‡ Nanoscale Solid−Liquid Interfaces, Helmholtz-Zentrum Berlin für Materialien und Energie GmbH, Schwarzschildstraße 8, Berlin 12489, Germany; § Institut für Weltraumforschung, DLR, Rutherfordstr. 2, Berlin 12489, Germany; ∥ Institut für Planetologie, 9185 Universität Münster, Wilhelm-Klemm-Str. 10, Münster 48149 Germany; ⊥ Institut für Physik und Astronomie, Technische Universität Berlin, Straße des 17. Juni 135, Berlin 10623, Germany

**Keywords:** infrared spectroscopy, nanoscopy, planetary materials, minerals, optical anisotropy

## Abstract

Nanospectroscopic investigations of the mineralogical composition of materials returned via sample-return missions are crucial for our understanding of the origin and evolution of planetary objects in our Solar System. Here, we show that the emerging technique of photothermal polarimetric nanoscopy, a variant of atomic force microscopy-based infrared spectroscopy, enables one to derive infrared fingerprint spectra of minerals noninvasively on the nanoscale. Besides the spatially resolved identification of specific minerals and mineral phases, the evaluation of the polarization dependence of the photoinduced nanomechanical response, in combination with optical reference data, may allow the deduction of valuable structural information on individual nanocrystallites or grains embedded in solid matrices.

## Graphical abstract

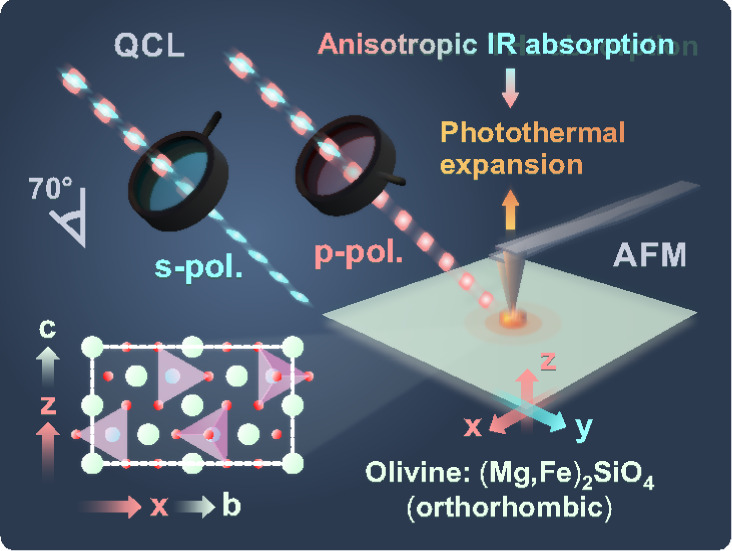

## Introduction

Studying minerals of extraterrestrial origin has been an important approach to unravel the geochemistry and, thereby, the origin and evolution of planetary bodies in our Solar System for more than two centuries. The first investigations of this type focused on the mineralogy of meteorites as early as the beginning of the 19th century^1^ and have since identified the presence of more than 250 different mineral species, reflecting diverse environments and formation conditions.^2^ Optical spectroscopy, in particular vibrational spectroscopy techniques such as infrared spectroscopy or Raman spectroscopy, has played an important role since its emergence as an established research tool in the mid-20th century. Spectroscopy in the long-wavelength range, from the infrared to the THz range, has been employed successfully for the investigation of planets, moons, asteroids, or interstellar matter via remote sensing (see, e.g.,^3–5^ ) and, already very early on, for studying material returned from the Moon.^6^ Sample return missions are becoming increasingly feasible and have, in the meantime, successfully returned samples from beyond Earth’s orbit providing material from the solar wind,^7^ the comet Wild 2,^8^ and the asteroids Itokawa^9^ and Ryugu.^10^ Optical techniques, in particular those sensitive to the vibrational fingerprint, which enable noninvasive access to structural and compositional properties beyond what can be derived from remote sensing or robotic explorations, are therefore in very high demand. Because of the heterogeneous nature of many planetary materials, Raman or infrared microspectroscopy has frequently been employed (see, e.g., refs 10,11 ), and there are some examples of employing nanospectroscopy techniques such as infrared scattering near-field optical microscopy (IR-SNOM)^12^ and atomic force microscopy-based infrared spectroscopy (AFM-IR).^13–16^

Surface planetary materials are often fine-grained. They form heterogeneous superficial deposits, often referred to as regolith, which are typically affected by strong physical and chemical weathering processes^11^ and are commonly dominated by submicron grains. Small grains embedded in large solid matrices can hold crucial information about the origin and age of celestial bodies. Mesoscale data on the inclusion structure, e.g., measured as mean grain orientation, can be used for placing constraints on metamorphic history or impactors, for example, in the characterization of chondritic meteorites.^17^ For fine regolith particles, such as those returned by the space missions to near-Earth asteroids, the accurate combined microstructural and chemical resolution of individual microcraters and inclusions (with typical sizes <1 μm) has revealed important insights into the the interaction of solar system asteroids with interplanetary dust.^18^

The aim of this study is to investigate whether polarization-resolved AFM-IR or photothermal polarimetric nanoscopy (PPN), respectively, is a suitable approach to study the structure and composition of minerals on the nanoscale and, hence, can represent a complementary technique to infrared and Raman microspectroscopies in planetary and Earth sciences.

In the AFM-IR method, the AFM tip probes the local photothermal expansion of a sample upon laser radiation. The spatial resolution of AFM-IR significantly surpasses the diffraction-limited lateral resolution of classical far-field optical experiments. Typical lateral resolutions are in the range of about 10–30 nm and depend on a complex interplay of parameters, such as the measurement mode, the probing depth, the laser pulse rates, thermal conductivity, and interfacial thermal resistance.^19–21^ The method is an “in-depth probing technique”^21^ which can probe depths of a few hundred nanometers^21,22^ to a few micrometers at frequencies with low absorption.^22^ By adapting pulsing parameters, the surface selectivity can be improved.^23^

Adding a polarization control allows probing anisotropic absorption of organic and inorganic samples at the nanoscale.^24^ Dazzi et al. showed that the measured AFM-IR signal relates to the sample absorbance in cases where the variation of the refractive index is small^25^ and, in general, relates to the imaginary part of the dielectric function.^24,25^ In AFM-IR measurements, the anisotropic absorption properties of thin films or bulk materials are caused by the anisotropic dielectric function and, e.g., preferential directions of transition dipole moments along defined axes. However, for thin films and structured surfaces, anisotropic absorption can also be induced by anisotropic contributions of field intensities.^24^

Different olivine samples with varying levels of complexity are studied. Analytical challenges include the varying sizes of anisotropic crystalline olivine inclusions (ranging from nanometers to micrometers) within a surrounding matrix. To show the principle’s applicability, a natural olivine single crystal is studied first. Afterward, the method is applied to the investigation of olivine samples with increasing complexity, olivine nanograins in a multicrystalline sample and a few 10 μm-sized inclusions in a glass matrix.

## Experimental Section

Olivine is a common mineral phase in all rocky planetary environments. Furthermore, planetary materials tend to be very fine-grained on submicron scales. Therefore, olivine in matrices serves as an ideal starting point.^26^

### Olivine Samples

Two natural olivine samples, provided by the Institute for Geosciences at the University of Jena (Germany), were investigated in this work.^27^

The homogeneous forsterite (Fo90.5) single crystal, with a polished surface and the *c*-axis oriented normal to it (parallel to z), was aligned with the plane of incidence (*xz*-plane in all experiments) so that the *a*- and *b*-axes were parallel to *y* and *x*, respectively, see Figure 1. The multicrystalline sample with slightly different forsterite content (Fo91.3), included differently oriented crystalline domains.^27^

**Figure 1:**
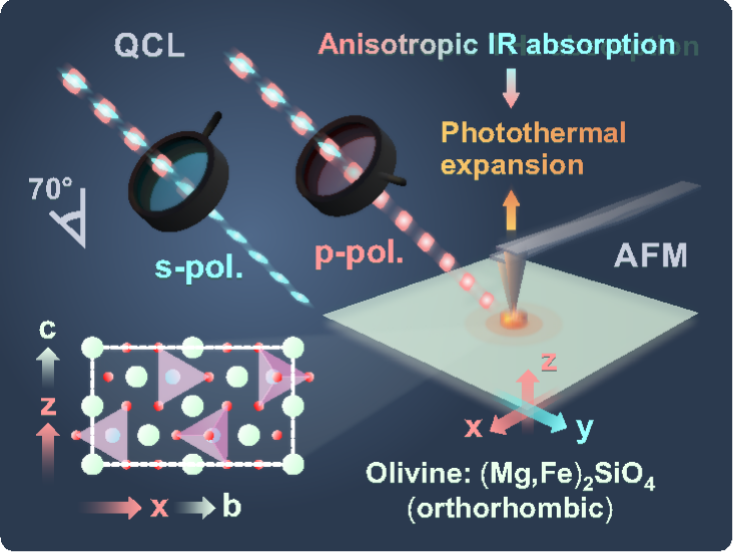
Fundamental principle of photothermal polarimetric nanoscopy (PPN) and experimental geometry in the study of the single-crystal olivine sample. PPN is based on the evaluation of the polarization of atomic force microscopy-based infrared spectroscopy (AFM-IR).

Both samples were regularly cut and polished using lapidary techniques to prepare minerals for optical studies.

In detail, the samples were embedded in epoxy resin, and the block was cut with a diamond saw. The selected halves were fixed on a sample holder on a specialized lapping/polishing machine to ensure homogeneous polishing and an entirely flat and planar surface without any curvature.

The sample surface was polished using progressively finer abrasives (diamond paste in suspension) on a polishing cloth to ensure optical quality, with the final grain size of 0.25 μm.

### Experimental Setup

The PPN measurements^24^ were performed using a commercial AFM-IR platform (nanoIR2-FS, Anasys Instruments/BRUKER) equipped with an external cavity quantum cascade laser (QCL) (MIRcat, Daylight Solutions) in a top down geometry. The setup was mounted on a vibration isolation table. Experiments were conducted under ambient conditions, i.e., at room temperature and a stable humidity level below 2%, controlled by dry air purging. Measurements were performed in contact mode using gold-coated silicon AFM tips (0.07–0.4 N/m spring constant) with a nominal radius of curvature of the tip apex ≈30 nm, serving as a benchmark for the lateral resolution of the AFM-IR method.^13^ Switching the polarization state (*p*- to s-polarized and vice versa) of the intrinsically linearly polarized QCL pulses (100:1 extinction ratio) was achieved by implementing an automated polarization rotator consisting of a series of flat Au mirrors. QCL light with a spectral range of 900–1125 cm^–1^ and an average power of less than 0.45 mW (olivine single crystal), 0.91 mW (nanocrystallite), and 0.1 mW (microcrystallite) was focused under the tip with a spot size of about 50 μm × 17 μm at an angle of incidence to the surface normal of 70°. For the chosen settings, a local temperature rise of a few K is possible; about 6 K was expected in a study of silica glasses in the center of the IR beam (2 mW, 100 s pulse width).^22^ For such temperature rises in the range of a few K, damage to the studied samples is not expected.

The tunable QCL pulse rate was synchronized with one-third (197 kHz for the olivine single crystal, 224 kHz for nanocrystallite, and 134 kHz for the microcrystallite) of the low-noise fourth bending mode of the AFM cantilever in contact with the sample. The resonant cantilever oscillation signal, collected by the AFM deflection detection system, was filtered using a bandpass filter with a window of 50 kHz around the central frequencies (591, 672, and 403 kHz, respectively). The photothermal expansion measurements were normalized to the corresponding polarization-dependent QCL background collected by the IR detector before focusing on the sample. The mechanical resonance enhancement of the cantilever oscillation amplitude allows for high sensitivity of the method, down to ultrathin films and monolayers.^28–30^ A spectral resolution of 1 cm^–1^ was accessible at a 20 cm^–1^/s QCL sweep rate, corresponding to an an acquisition time of 11.3 s per spectrum, currently limited by the external cavity grating stabilization time of the QCL. Savitzky–Golay filtering (third order, five points) was applied to the spectral data. AFM height images (500 × 300 pixels) were measured using a scan rate of 0.40, 0.25, and 0.2 Hz per line (for olivine single crystal, nanocrystallite, and microcrystallite, respectively). The acquisition time for single-wavenumber photothermal expansion images of olivine nanocrystallite was therefore 20 min per image, currently limited by the AFM scan rate. Only the data in the retrace scan direction were collected, i.e., the stage moving in the *x*-direction toward the laser source (see Figure 1). The images were flattened (first order) using the built-in nanoIR2-FS software, Anasys Studio 3.12.

## Results

### Olivine Single Crystal

Figure 2 shows, at the top, an AFM image as well as averaged s- and p-polarized PPN spectra of a natural olivine single crystal (Mg_1.9_Fe_0.1_SiO_4_) sample. The probed measurement spots are marked. Bands in the PPN spectra are related to the anisotropic SiO_4_ stretching vibrations (peak 1 at about 960 cm^–1^; peak 2 at about 983 cm^–1^) and to the Christiansen feature (*n* = 1 effects, see peak 3 at about 1040 cm^–1^ and peak 4 at about 1065 cm^–1^).

**Figure 2:**
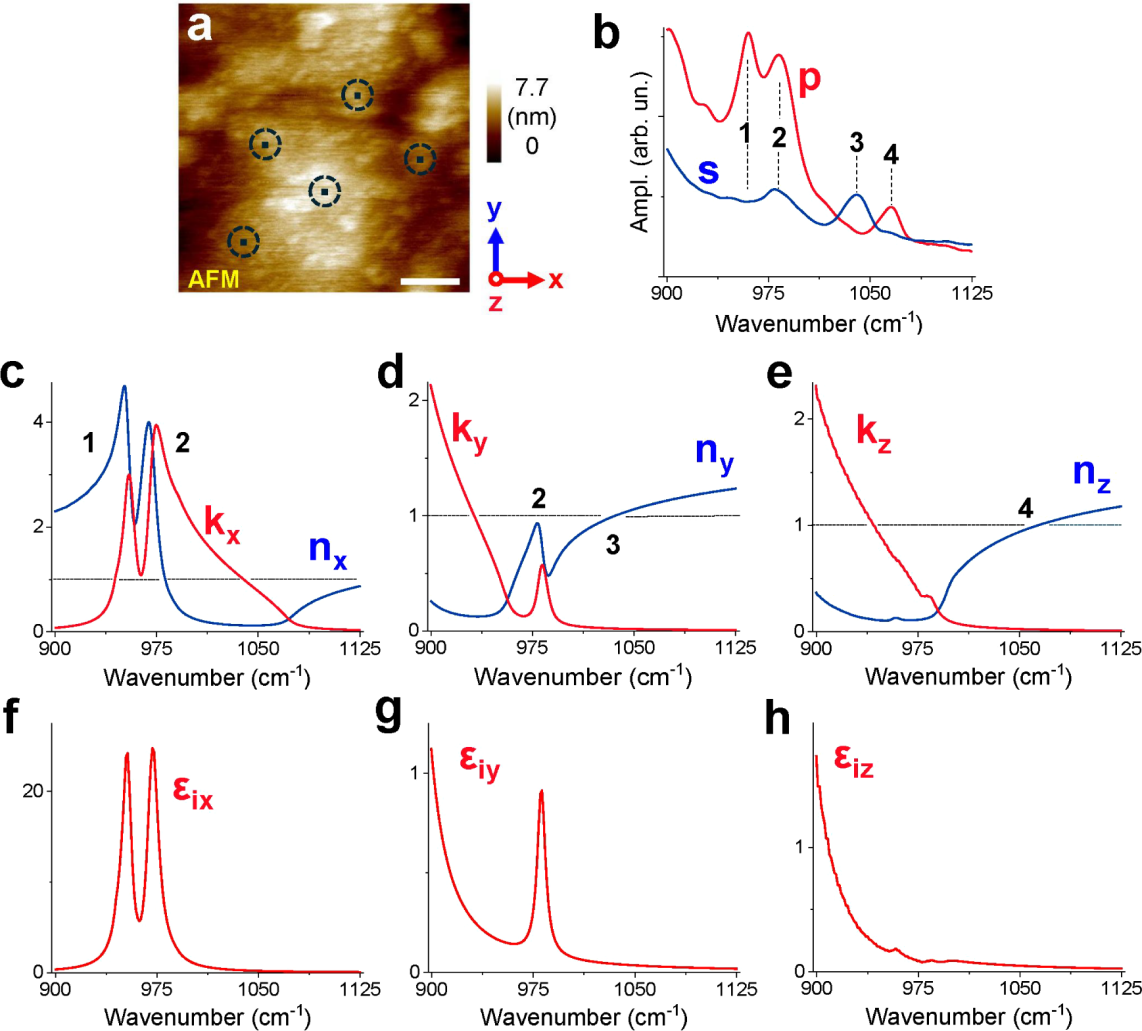
AFM image of a single-crystal olivine sample: (a) Average of PPN spectra taken for s- and p-polarized QCL beams at the indicated positions. Scale bar is 200 nm (b) and anisotropic optical constants as taken from ref 31 (c–e). On bottom the respective imaginary parts of epsilon are shown (f–h). Reference *n* and *k* data of a natural olivine single crystal (Mg1.9Fe0.1SiO4) along the *b*-, *a*-, and *c*-axes correspond to the indicated *x*-, *y-*, and *z*-axes of the sample, respectively. Adapted with permission from ref. 31. Copyright 2001 EDP Sciences.

In the measurement geometry used at a 70° incidence angle, the p-polarized radiation has field components in *x*- and *z*-directions of the sample (Figure 1), and therefore, the measured p-polarized photothermal signal is proportional to the sum of the imaginary parts of the dielectric function in these directions. In contrast, the s-polarized radiation only has field components in the *y*-direction of the sample and, therefore, can only be related to the imaginary part of the dielectric function in *y*-direction. From the comparison with literature reference data^31^ of the direction-dependent optical constants (*n*, *k*) in Figure 4c–e, we confirm that the *x*, *y*, *z*-axes of the olivine single crystal sample are parallel to the crystallographic *b*-, *a*-, and *c*-axes, respectively. In particular, the bands observed in the measured s-polarized spectrum in Figure 2b can only be related to the olivine single crystal optical constants in the *y*-direction (*a*-axis).

The measurements of the single crystal exemplarily show that, similar to the analysis of organic films, conventional AFM-IR enables fingerprinting and that PPN, furthermore, can provide access to information about the orientation of the investigated crystals.

### Nanograin in Multicrystalline Olivine

Figure 3a displays an AFM image of a multicrystalline olivine sample. A point marks the spot that was selected for the subsequent PPN spectra and image acquisition. Peaks 3 and 4 associated with the Christiansen effect (*n* = 1 of the *a*- and *c*-crystal axes), appear in either the s- or p-polarized spectrum in a similar order as for the single crystal (Figure 2b). However, unlike the single crystal, peaks 1 and 2 are observed for both the s- and p-polarized PPN spectrum (Figure 3b). From this finding, a different crystal axis orientation compared to the olivine single crystal (Figure 2) can be concluded. A further quantitative determination of the orientation is, in principle, possible but is beyond the scope of this article. It would require detailed research of the Christiansen features in dependence on the azimuthal rotation, an optical simulation of the direction-dependent absorption properties in the range of the vibrational bands, and a quantitative understanding of the correlation between photothermal expansion and the absorption properties in dependence on the penetration depths of radiation.

**Figure 3:**
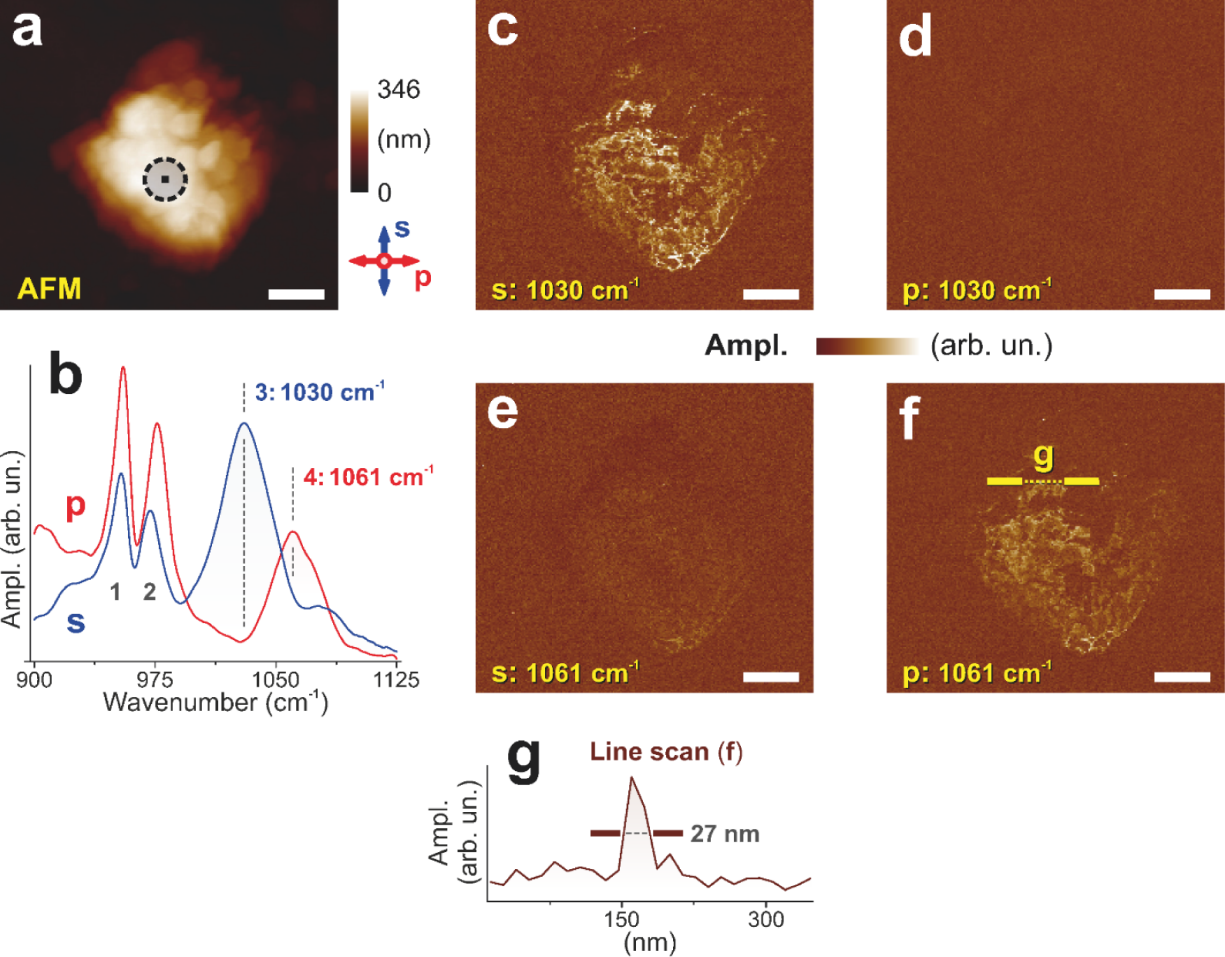
PPN measurements of a multicrystalline olivine sample. Scale bars are 500 nm. (a) AFM image with marked points selected for polarization-dependent spectra acquisition. (b) s- and p-polarized PPN spectra. (c–f) s- and p-polarized PPN maps at the frequencies of peaks 3 and 4 of the nanocrystallite (left to right and top to bottom, respectively). (g) Demonstrates the 27 nm lateral resolution of the method.

The frequencies of the 3,4 peaks are chosen for further s- and p-polarized imaging (Figure 3 c–f). It can be seen that the nanosized crystal grain crystallite is well separated from the surrounding olivine because of the different AFM cantilever bending mode frequencies in these two areas. The PPN signal along a selected line (yellow, dotted, Figure 3f) is presented in Figure 3g.

In summary, the results prove that phenomenological fingerprints and orientation information can be achieved on the nanoscale at sub-50-nm resolution.

### Olivine Inclusions in a Glass Matrix

In Figure 4b, the s- and p-polarization-dependent PPN spectra of an olivine inclusion in a glass matrix are displayed. The positions of characteristic bands (1 at 960 cm^–1^; 2 at 980 cm^–1^; 3 at 1033 cm^–1^) are close to the positions observed previously for the olivine single crystal (Figure 2) and the multicrystalline sample (Figure 3). The different spectral signatures in the s- and p-polarized spectra indicate, once again, an anisotropic crystalline structure. The broad background observed can tentatively be assigned to the contribution of the glass matrix (see comparative measurements in Figure 4f).

**Figure 4:**
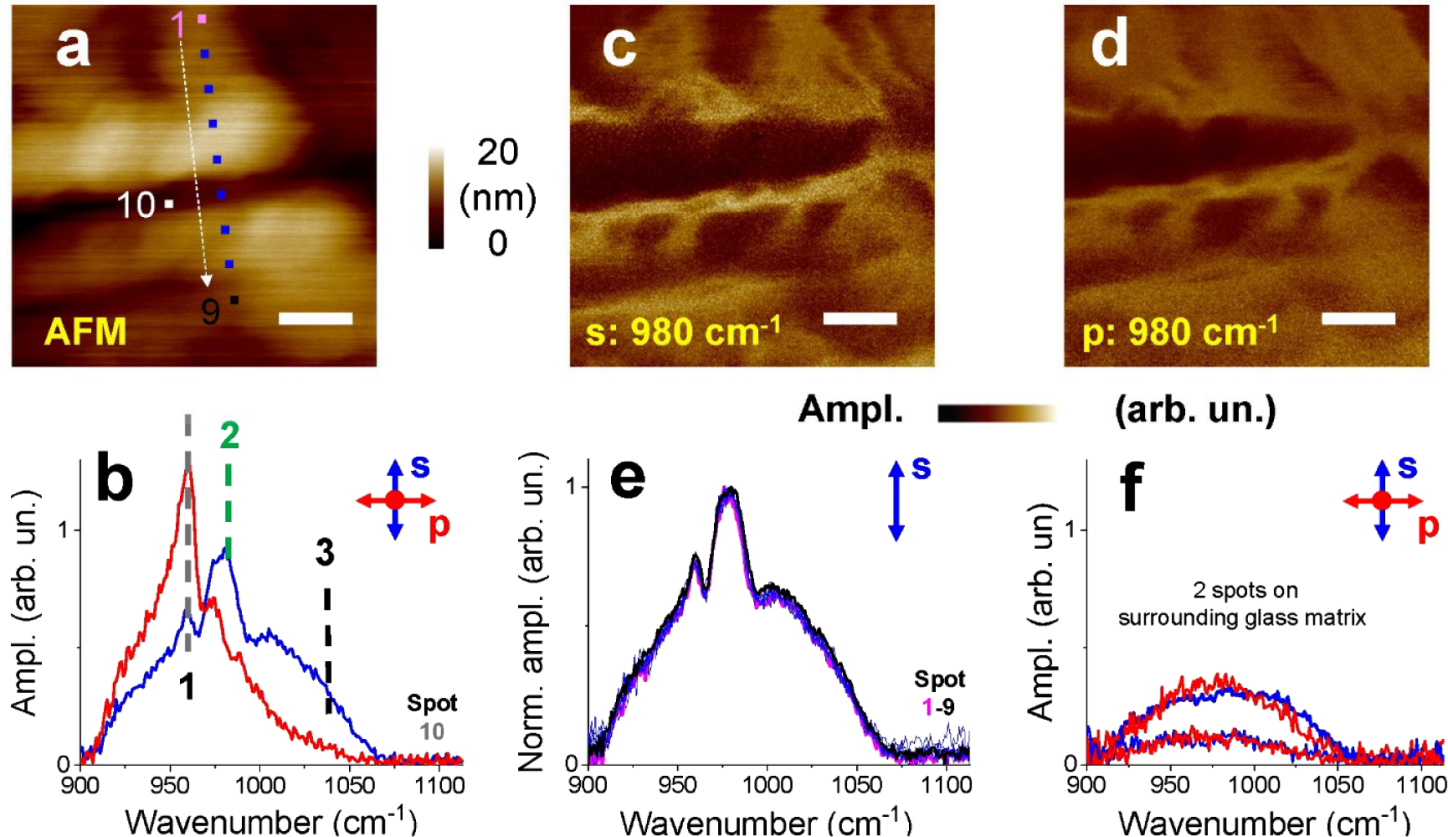
PPN measurement of a few 10 μm large olivine crystallites embedded in a glass matrix. (a) AFM measurement; scale bar = 100 nm. The 10 spots measured by PPN are indicated. (b) s- and p-polarized PPN spectra at position 10. (e) Normalized s-polarized PPN spectra along one line from positions 1 to 9. (c, d) PPN maps of the sample at the frequency of peak 2 (980 cm^–1^) and (f) two exemplarly s- and p-polarized PPN spectra at two positions on the surrounding amorphous glass matrix (outside of the range of the AFM images).

A map of the p-polarized IR amplitude at 980 cm^–1^ in Figure 4d (close to the maximum of band 2) correlates to some degree qualitatively with the morphology reflected by the AFM image in Figure 4a. Because changes in the sample stiffness or topography induce a shift in the resonance frequency,^21^ they can induce changes in the measured amplitude of the presented maps and be responsible for sharp features in the IR amplitude. They indicate changes of crystallinity and/or crystal orientation along what appear to be grain boundaries on a subnanometer level. To investigate possible spectral variations, a line of s-polarized PPN spectra was measured along 9 positions and is shown in Figure 4e.

Assuming a strict correlation between the spectra and structure, the normalized spectra in Figure 4e indicate a chemically homogeneous material for the measured single spots.

The results shown in Figure 4 prove that PPN measurements can clearly resolve the olivine crystallites in the synthetic glass matrix via the identification of characteristic spectral signatures of olivine. The polarization-dependent spectra prove an anisotropic nature of the crystallites; furthermore, there are indications that a broadband background arises from the surrounding glass matrix. However, as also discussed with respect to the previous example, further detailed studies beyond the scope of this paper are required to gain more quantitative information and will be part of future work.

## Conclusion

The applicability of PPN to achieve phenomenological fingerprint spectra and orientation information on olivine crystals and inclusions has been demonstrated. For a natural olivine single crystal, the orientation was determined by correlation to the literature optical constants. For all studied samples, features related to the Christiansen effect are also observed. These features appear at frequencies where *n* = 1 and are, due to the direction-dependent optical constants, highly indicative of the orientation of an anisotropic crystal. The PPN images indicate, for both the multicrystalline olivine and the olivine inclusion in a glass matrix, spatial changes in the optical response on the submicron level. These finely and spatially resolved features can often not be correlated to features observed in the morphology and hence show the added value of PPN and its high potential in planetary science applications.

Combined microstructural and chemical analyses can be particularly relevant for mineral grains in optically transparent extraterrestrial matrices. So far, a few-tens of μm-sized particles have typically been returned by space probes to solar system objects. Small inclusions in these particles are typically on the nanometer scale, both in size and in the distances between them.^18^ The individual chemical differentiation of grains in such assemblies requires mesoscale probing, which, as this study demonstrates, is enabled by PPN.

Macroscale crystal orientation can, in principle, also be accessed for most minerals with anisotropic lattice symmetries using polarization-resolved Raman microscopy; see, for example, the case of olivine.^32^ This also applies to many polymorphs. However, inherently low Raman scattering efficiencies imply highly focused beams, which can lead to overheating of micron-sized grains or inclusions trapped in matrices (up to several hundred degrees^33^) and to the alteration of unstable mineral phases.^34^ Another risk is photochemical alterations. On the contrary, PPN, operating at infrared wavelengths, avoids photochemistry altogether and merely induces heating of the irradiated areas by merely a few degrees^28^ and hence is noninvasive.

Table 1 shows a comparison of PPN with nanospectroscopic techniques that have, in the past, been employed to study mineral samples.

**Table 1:** Comparison of Nanospectroscopic Techniques Previously Employed to Study Mineral Samples

Technique	Infrared-active	Raman-active	Spatial resolution	Sensitive to crystal symmetry	Advantages	Disadvantages
**PPN** **(this work)**	x		10–30 nm	x	Access to vibrational fingerprint of minerals, orientation dependence	AFM tip in sample environment
**tip-enhanced** Raman^35^		x	<100 nm	x	Access to vibrational fingerprint of minerals, orientation dependence	AFM tip in sample environment, Photodamage risk, tip degradation
**nano** FTIR^12^	x		few 10 nm		Access to vibrational fingerprint of minerals	AFM tip in sample environment

It should be noted that, although the PPN technique is in this work demonstrated on the example of olivine, the findings are applicable to all minerals exhibiting vibrational resonances within the spectral range accessible to the PPN setup.

Finally, an extension of the PPN spectral range toward longer and shorter wavelengths would be favorable, as this would add to the chemical specificity of the technique, allowing access to a wider region of characteristic lattice and bending vibrational modes, as well as high-energy internal stretching vibrations. Intensive work on the availability of custom-made QCLs of suitable performance is in progress (see, e.g. ref 36).
